# A case report of anaphylactic shock caused by lidocaine

**DOI:** 10.1097/MD.0000000000041325

**Published:** 2025-01-24

**Authors:** Jiang-Hui Mi, Ting-Ting Shen, Hong-Wei Wang

**Affiliations:** a Shanxi Provincial Integrated TCM and WM Hospital, Taiyuan, China; b Shanxi University of Chinese Medicine, Shanxi, China.

**Keywords:** anaphylaxis, case report, epinephrine, lidocaine

## Abstract

**Rationale:**

Local anesthesia is a widely used technique for emergency wound closure, with lidocaine among the most commonly employed local anesthetics. Allergic reactions to lidocaine are rare, with anaphylaxis being even more uncommon.

**Patient concerns and diagnosis:**

This report describes a 72-year-old male patient who presented with a right foot injury and underwent wound suturing under lidocaine local anesthesia. Although the procedure went smoothly, the patient developed dizziness, cold sweats, hypotension, and bradycardia 30 minutes later, leading to a diagnosis of anaphylaxis due to lidocaine allergy.

**Interventions and outcomes:**

Immediate treatment for anaphylactic shock was initiated, including intramuscular administration of adrenaline, fluid resuscitation, anti-inflammatory agents, antihistamines, oxygen therapy, and symptomatic supportive care. Within 20 minutes of active treatment, the patient’s symptoms were effectively controlled. The patient was safely discharged after 24 hours of observation. Health education was provided to enhance self-management skills.

**Lessons:**

Although rare, anaphylaxis induced by lidocaine can be fatal. Early recognition, prompt intervention, thorough preoperative assessment, and careful postoperative monitoring are critical to improving survival rates.

## 1. Introduction

Anaphylaxis is a rapidand severe allergic reaction that can be life-threatening. According to the World Allergy Organization, anaphylaxis is characterized by typical skin symptoms and at least one additional systemic symptom, such as respiratory and/or cardiovascular symptoms, acute hypotension, bronchospasm, or laryngeal involvement.^[1]^ These reactions may occur even in the absence of typical skin manifestations and can develop within minutes to hours following exposure to a known or highly suspected allergen. The incidence of anaphylaxis varies by region, but the accepted incidence of severe anaphylactic reactions is estimated to be 1 to 3 per 10,000 people.^[1,2]^

Local anesthesia is a common technique used in emergency settings for procedures such as wound debridement and suturing. Lidocaine is one of the most frequently used local anesthetics, which works by inhibiting sodium ion influx into nerve cells, preventing depolarization, and blocking the conduction of pain signals.^[3]^ Although allergic reactions to lidocaine are extremely rare, they can occur, typically within 30 minutes of exposure.^[4]^ A study by Lopes et al found that the incidence of allergic reactions to lidocaine was less than 1% in a survey of 2978 patients,^[5]^ although the mortality rate can be high in severe cases.

This report details a case in which a patient developed anaphylactic shock following wound suturing under lidocaine local anesthesia.

## 2. Case report

A 72-year-old male patient was transferred from the emergency department to the internal medicine department due to dizziness and cold sweats lasting 3 minutes. His medical history included a cholecystectomy, during which he received general anesthesia with no reported adverse reactions. There was no known history of food or drug allergies.

**Figure 1.**
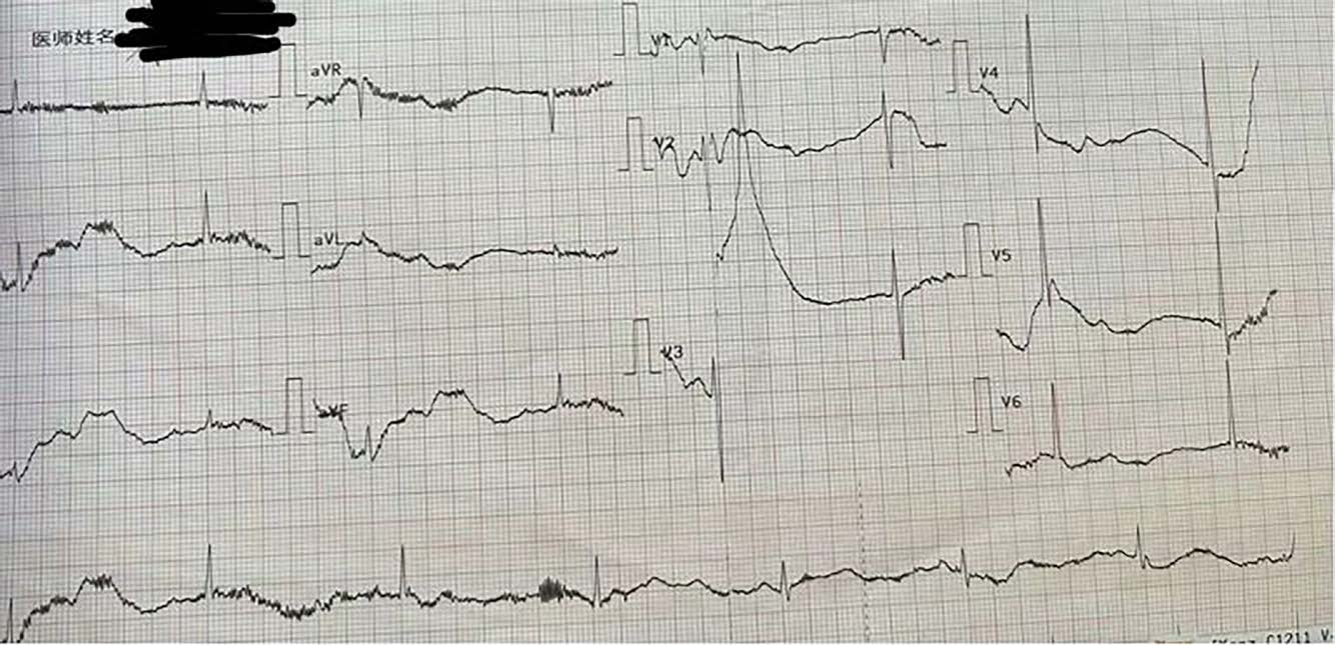
Electrocardiogram report of the patient before treatment.

On May 5, 2024, at 23:10, the patient presented with a 4 cm wound on his right foot. The orthopedic team performed wound debridement and suturing under local anesthesia with lidocaine at 23:15. A tetanus antitoxin skin test was administered at 23:30. Fifteen minutes later, the patient experienced dizziness, palpitations, and profuse sweating. His blood pressure was recorded at 61/34 mm Hg (low), and his heart rate was 42 beats per minute during the episode. Electrocardiogram findings revealed significant sinus bradycardia and an abnormal T wave, which was shown in Figure 1. The patient’s fingertip blood glucose was measured at 6.8 mmol/L. Based on the clinical presentation and the definition of anaphylactic shock, which includes hypotension following exposure to an allergenic drug, a diagnosis of anaphylactic shock was made. Laboratory results were within normal limits in Table 1.

**Table 1:** The significant laboratory test results at the first admission

Inspection item	Values	Unit	Reference range
Temperature	36.4	°C	36.0–37.0
Blood pressure	61/34	mm Hg	90/60–140/90
Heart rate	42	times/min	60–100
Respiratory frequency	26	times/min	12–20
PH	7.368		7.35–7.45
PO_2_	131.4	mm Hg	80–100
PCO_2_	35	mm Hg	35–45
BE	−4.8	mmol/L	–3–3
GLU	8.16	mmol/L	3.9–6.1
K	3.84	mmol/L	3.5–5.3
CK-MB	8.8	U/L	0–24
CK	20	U/L	24–170
CTN-I	0.022	ng/mL	0–0.3
Cr	65.1	μmol/L	59–104
WBC	11.52	10^9^/L	3.5–9.5
NEU%	49.40	%	40–75
HGB	134.00	g/L	130–175
PLT	219.00	10^9^/L	125–350
D-D	0.80	mg/L	0–1.5

CK = creatine kinase, CK-MB = creatine kinase; MB Form, Cr = creatinine, CTN-I = troponin. Troponin Troponin I, D-D = D-dimer, GLU = lood lucose, HGB = hemoglobin, K = potassium determination, NEU% = Neutrophil percentage, PCO2 = Partial Pressure of Carbon Dioxide, BEbase excess, PH = hydrogen potential, PLT = Platelet, PO2 = Partial Pressure of Oxygen, WBC = White Blood Cell.

## 3. Therapeutic intervention

Immediate treatment was initiated following the 2020 anaphylaxis management guidelines from the World Allergy Organization.^[1]^ Epinephrine (0.1 mg, 1:1000) was injected intramuscularly into the lateral quadriceps femoris.^[6]^ The patient was positioned supine and administered high-concentration oxygen. Continuous electrocardiogram monitoring was performed, and two intravenous lines were established for fluid resuscitation. The patient was also treated with the following medications:

- **Epinephrine (0.1 mg, IM)**–First-line treatment for controlling allergic reactions
- **Diphenhydramine (10 mg, IM)**–To alleviate allergic symptoms (administered as an antihistamine)
- **Dopamine (10 mg, IV)**–To elevate blood pressure
- **Atropine (0.5 mg, IV)**–To address bradycardia^[7]^

**Figure 2.**
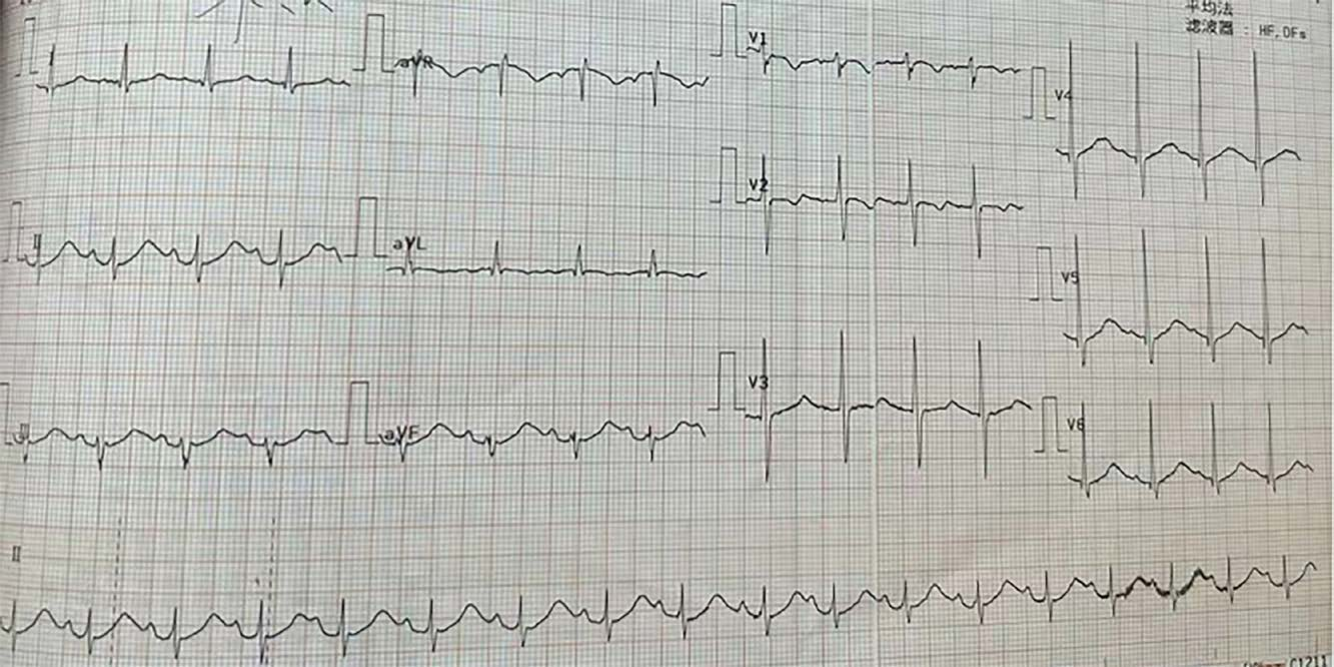
Electrocardiogram report of the patient after treatment.

The patient’s vital signs were closely monitored, and within 10 minutes, his blood pressure stabilized around 112/70 mm Hg, and his heart rate increased to 95 beats per minute (Fig. 2). Limb tremors were observed, likely due to residual histamine release. These were treated with hydrocortisone, calcium gluconate, and vitamin C.

After 24 hours of observation in the emergency department, the patient was discharged in stable condition. He was instructed to take cetirizine (10 mg) orally once daily, avoid spicy and stimulating foods, and refrain from consuming high-protein foods (e.g., tropical fruits, beef, lamb, and seafood). The patient was also advised to wear a mask when going out and to follow up for allergen testing after discontinuing antihistamines for 7 days.

## 4. Discussion

The patient had no prior history of food or drug allergies and had not been exposed to known allergens such as seafood, peanuts, or peaches. On the day of presentation, only local anesthesia with lidocaine and a tetanus antitoxin skin test were administered. No local reactions were observed at the injection site of the tetanus antitoxin. Given the absence of other possible triggers, anaphylactic shock due to lidocaine allergy is highly suspected. Literature review reveals reports of lidocaine-induced allergies, and forensic autopsy findings from some sudden death cases suggest that anaphylactic shock due to lidocaine was the cause of death.^[8,9]^ Therefore, this case is most likely an instance of anaphylactic shock induced by lidocaine.

This case highlights the importance of early recognition and management of anaphylaxis induced by lidocaine, a rare but potentially fatal event. Although rare, anaphylactic reactions to lidocaine can occur within minutes to half an hour of exposure. Therefore, it is essential to monitor patients closely after the administration of local anesthetics. Immediate administration of epinephrine remains the standard treatment for anaphylactic shock, with intramuscular injection in the quadriceps femoris being the preferred route.^[6]^

Surgeons should inquire about a patient’s allergy history before administering lidocaine for local anesthesia.^[10]^ Post-procedural observation for at least 30 minutes in a clinical setting is recommended to detect early signs of anaphylaxis. Additionally, there is a need for more rapid allergy testing to identify patients at risk of lidocaine hypersensitivity prior to surgical procedures.

Interestingly, the patient in this case developed symptoms of tremors, which could be related to histamine release, a phenomenon that has been associated with allergic reactions.^[11]^ Moreover, while lidocaine is typically the suspected cause of allergic reactions, it is important to consider potential interactions with other drugs, such as tetanus antitoxin in this case. The possibility of an interaction between tetanus antitoxin and lidocaine warrants further investigation.

## 5. Conclusion

Anaphylaxis induced by lidocaine is rare but can be life-threatening. Early identification and prompt treatment with epinephrine are crucial for survival. Careful pre-procedural screening for allergies and post-procedural observation are crucial for preventing such adverse reactions. Furthermore, more research is needed to understand potential drug interactions and to develop rapid allergy testing methods for clinical use.

## Author contributions

**Conceptualization:** Hongwei Wang.

**Investigation:** Hongwei Wang.

**Methodology:** Hongwei Wang.

**Resources:** Jianghui Mi.

**Supervision:** Jianghui Mi.

**Writing – original draft:** Tingting Shen.

**Writing – review & editing:** Jianghui Mi.

## Supplementary Material


