# Supplementary material for: A case report of anaphylactic shock caused by lidocaine
Source: Medicine (Baltimore). 2025 Jan 24;104(4):e41325. doi: 10.1097/MD.0000000000041325 (PMC11771588; doi:10.1097/MD.0000000000041325)

**Informed consent for publication of clinical cases**

**Patient's name:** Yulin Zhang

**Article Title:** A case report of anaphylactic shock due to lidocaine

I am Yulin Zhang, consent to the publication of my photographs (omitting or obscuring identifying information) and medical records in an online format for publication and publicity in a journal. I have read the manuscript, understand the general description of what it contains, and have reviewed all photographs and illustrations involving me that will be published, and know that my name will not be published, and that the article may be re-published in other media after it is published in the casebook, and that it may be freely redistributed for any lawful purpose, including scholarly communication, translation, and commercial use.

**Patient Signature:**

**ID number.**

**Contact phone number:**

**Signed by the author:**

**ID** **number:**

**Contact phone number:**

Date: July 26, 2024


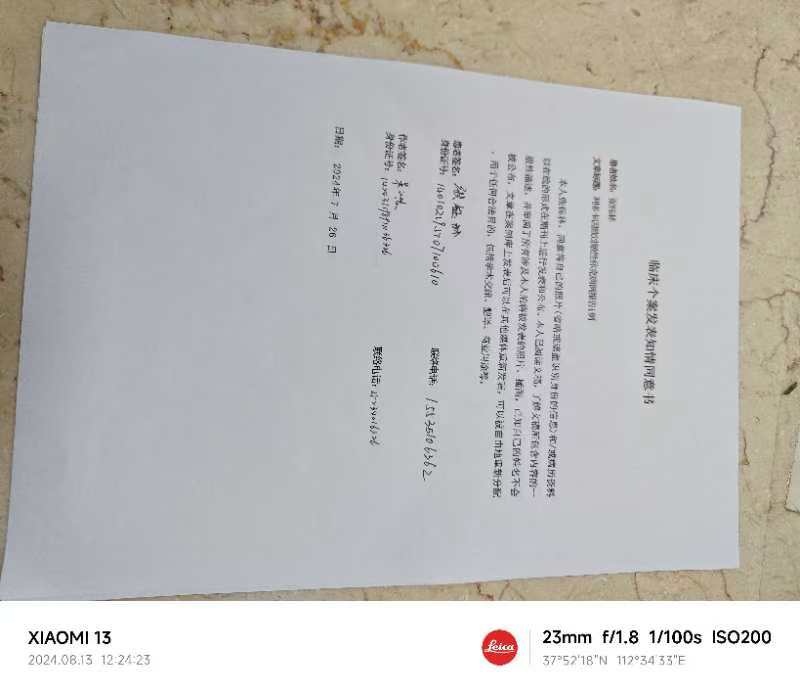

Supplement: Supplementary file 1 [file medi-104-e41325-s001.docx]
